# Supplementary material for: Critical evaluation of deep neural networks for wrist fracture detection
Source: Sci Rep. 2021 Mar 16;11:6006. doi: 10.1038/s41598-021-85570-2 (PMC7971048; doi:10.1038/s41598-021-85570-2)
Supplement: Supplementary file 1 — Supplementary Information. [file 41598_2021_85570_MOESM1_ESM.pdf]

Critical Evaluation of Deep Neural Networks for Wrist Fracture Detection: Supplementary Information

Abu Mohammed Raisuddin[1, +, *], Elias Vaattovaara[1,2,+,], Mika Nevalainen[1,2], Marko Nikki[2], Elina Järvenpää[2], Kaisa Makkonen[2], Pekka Pinola[1,2], Tuula Palsio[1,4], Arttu Niemensivu[1], Osmo Tervonen[1,2], and Aleksei Tiulpin[1,2,3]

[1]University of Oulu, Oulu, Finland
[2]Oulu University Hospital, Oulu, Finland
[3]Ailean Technologies Oy, Oulu, Finland
[4]City of Oulu, Oulu, Finland
[*]abu.raisuddin@oulu.fi
[+]these authors contributed equally to this work

S1 Dataset statistics

Dataset	Label	Sex	Count	Mean Age	SD of Age	Age Range	Number of Age Records
Training set	Fracture	Male	252	48.23	18.51	15 - 89	206
		Female	696	60.51	17.04	15 - 94	585
		Unknown	5	47.50	13.94	27 - 66	4
	Normal	Male	399	42.21	17.89	16 - 88	300
		Female	588	45.23	17.37	15 - 96	465
		Unknown	6	35.50	20.52	22 - 71	4
Test set #1	Fracture	Male	22	50.45	21.43	18 - 84	22
		Female	105	64.21	16.58	22 - 93	104
		Unknown	2	62.50	7.50	55 - 70	2
	Normal	Male	35	43.51	19.96	19 - 92	35
		Female	42	56.07	23.19	19 - 96	42
		Unknown	1	20.00	0.00	20 - 20	1
Test set #2	Fracture	Male	13	48.75	15.71	23 - 72	8
		Female	7	53.80	17.42	20 - 70	5
		Unknown	0	0.00	0.00	0 - 0	0
	Normal	Male	48	36.24	18.02	17 - 80	33
		Female	32	55.19	16.46	23 - 88	26
		Unknown	5	53.00	2.00	51 - 55	2

Table S1. Dataset Statistics. SD stands for Standard Deviation. The 'Number of Age Records' column indicates the number of cases for which the age data is recorded.

S2 Landmark Localization

Annotations for the landmark localization were annotated by the first author. For each radiographs three anatomical landmarks were annotated. These landmarks are: top of distal ulna, top of distal radius and assumed center of the wrist for PA view and

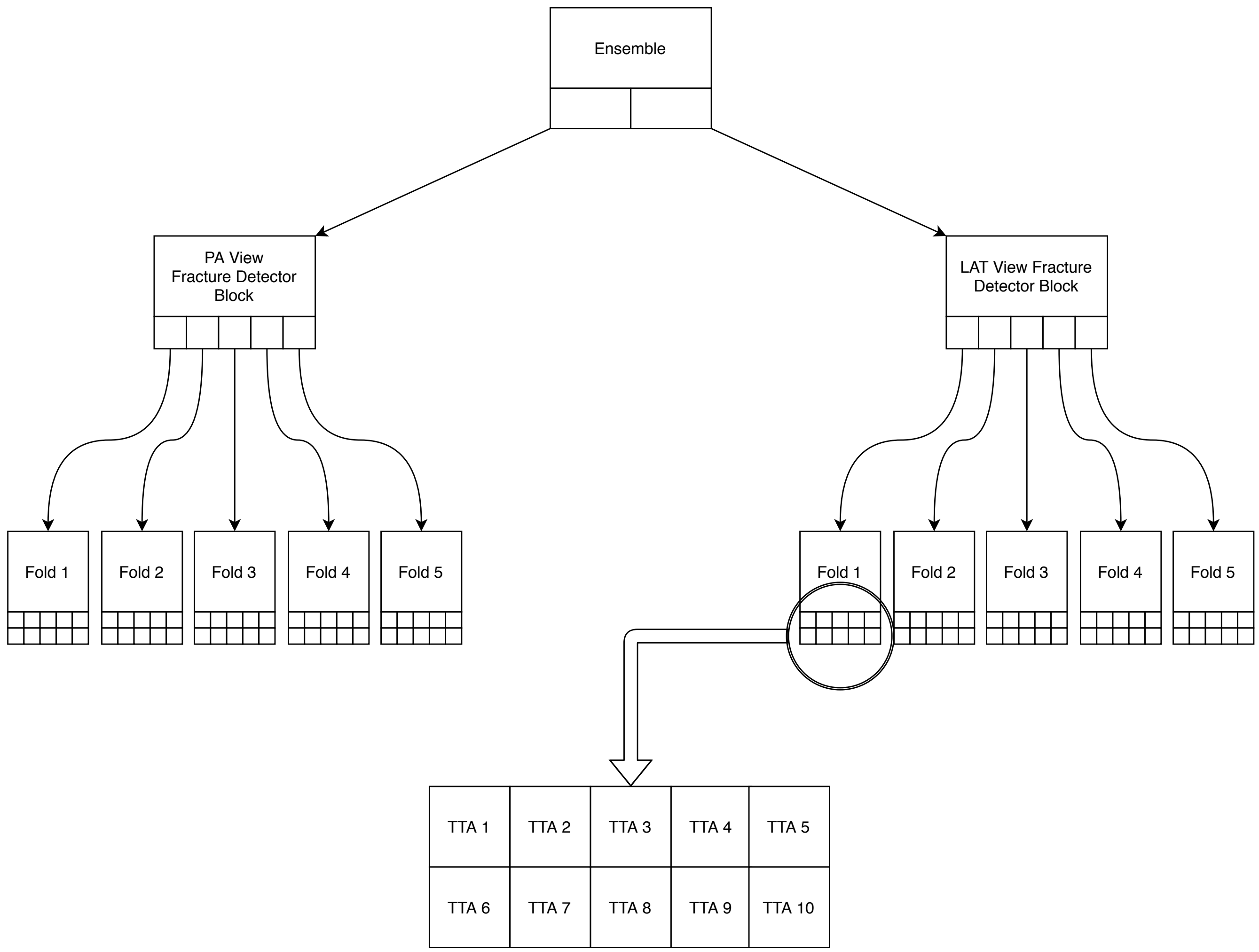

Figure S1. Structure of Both view ensemble of Fracture Detector Block. TTA stands for Test-Time Augmentation, PA for Posterioanterior, LAT for Lateral

two distinguishable landmarks on top part of distal radio-ulna and the assumed center of wrist for LAT view. Since these landmarks are not exact points we did intra-rater repeatability analysis. To that end, we randomly chose 100 radiographs from fracture and normal category for both PA and LAT view totaling 400 radiographs. Then we re-annotated them without assessing how they are annotated in the first annotation. Since it is not classification, we cannot compute the Cohen's Quadratic Kappa, instead, we calculated the recall at certain precision. With respect to first annotations, the second annotations scored recall of $0.16\,(0.12-0.19)$ at $2mm$ precision, $0.55\,(0.50-0.60)$ at $4mm$ precision, $0.70\,(0.65-0.74)$ at $5mm$ precision. If we calculate recall for X-coordinates only, we got a recall of $0.98\,(0.97-0.99)$ at $5mm$ precision and for Y-coordinates we got a recall of $0.87\,(0.84-0.90)$ at $5mm$ precision.We visualize the Precision-Recall curve for the landmark localizer in Figure S2.

S3 Inter-Rater Agreement Analysis

Test Set #1 Figure S3 shows inter-rater analysis using Cohen's Quadratic Kappa against the ground truth and the PCP1. Figure S4 and Figure S5 shows all against all agreement.

S4 Out-of-Distribution Experiment

Initially, we assumed that there is a distribution shift between general population cases and challenging cases of wrist fracture. If true, this could indicate that the general population cases are in-distribution or in-domain data and challenging cases are out-of-distribution (OOD) data. Thereby, the performance could be improved if we would add the data from Test Set #2 to the train set.

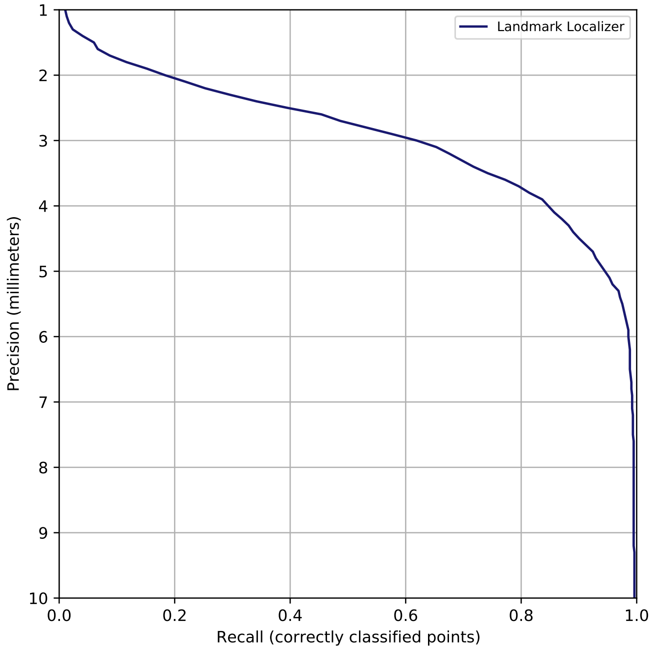

Figure S2. Precision-Recall Curve for Landmark Localizer

Recent works on uncertainty estimation (for example by Lakshminarayanan et al. work[1]) show that it is possible to detect OOD data samples using uncertainty estimates. To that end, we set up four Ensembles with 3, 5, 7, and 9 models, respectively. We did not use any cross validation for training Deep Ensemble, rather we split the whole training set into training and validation set and trained the Fracture Detection Block of our DeepWrist pipeline with different random initialization. Because of this, the approach Deep Ensemble lacked the ability to make use of transfer learning (as was done for the main model in the paper). We note that sole purpose of this experiment was to show that the challenging cases are not OOD data. The models for the ensembles were trained similarly to the main model shown in the paper, except, we did not use mixup.

We used Entropy and Predictive Variance as the estimated uncertainty of the corresponding prediction and used them to detect OOD samples. To obtain well calibrated uncertainty estimate, we calibrated the temperature of the models using the work of Guo et al.[2]. In Figure S6, we show AUROC and AUPR performance of OOD detection with Entropy and in Figure S7, we show the same with Predictive Variance. It is evident from AUROC and AUPR that the OOD detection performance is poor. Table S2 shows OOD detection AUROC with 95% confidence interval for different ensemble settings. Figure S8 shows the entropy distribution of in-domain (general population cases) vs OOD (challenging cases) data. Clearly, there is no noticeable shift in these entropy distribution. Considering, all the AUROCs, AUPRs and the entropy distribution, we can conclude that the Deep Ensemble cannot differentiate between general population data and challenging data well.

**# models**	**AUROC for OOD Detection (95% CI)**	
	Entropy based	Predictive Variance based
3	0.67 (0.61 - 0.73)	0.61 (0.55 - 0.68)
5	0.67 (0.61 - 0.73)	0.60 (0.54 - 0.66)
7	0.67 (0.61 - 0.73)	0.61 (0.55 - 0.67)
9	0.67 (0.61 - 0.73)	0.62 (0.56 - 0.68)

Table S2. AUROC of Deep Ensemble for OOD detection

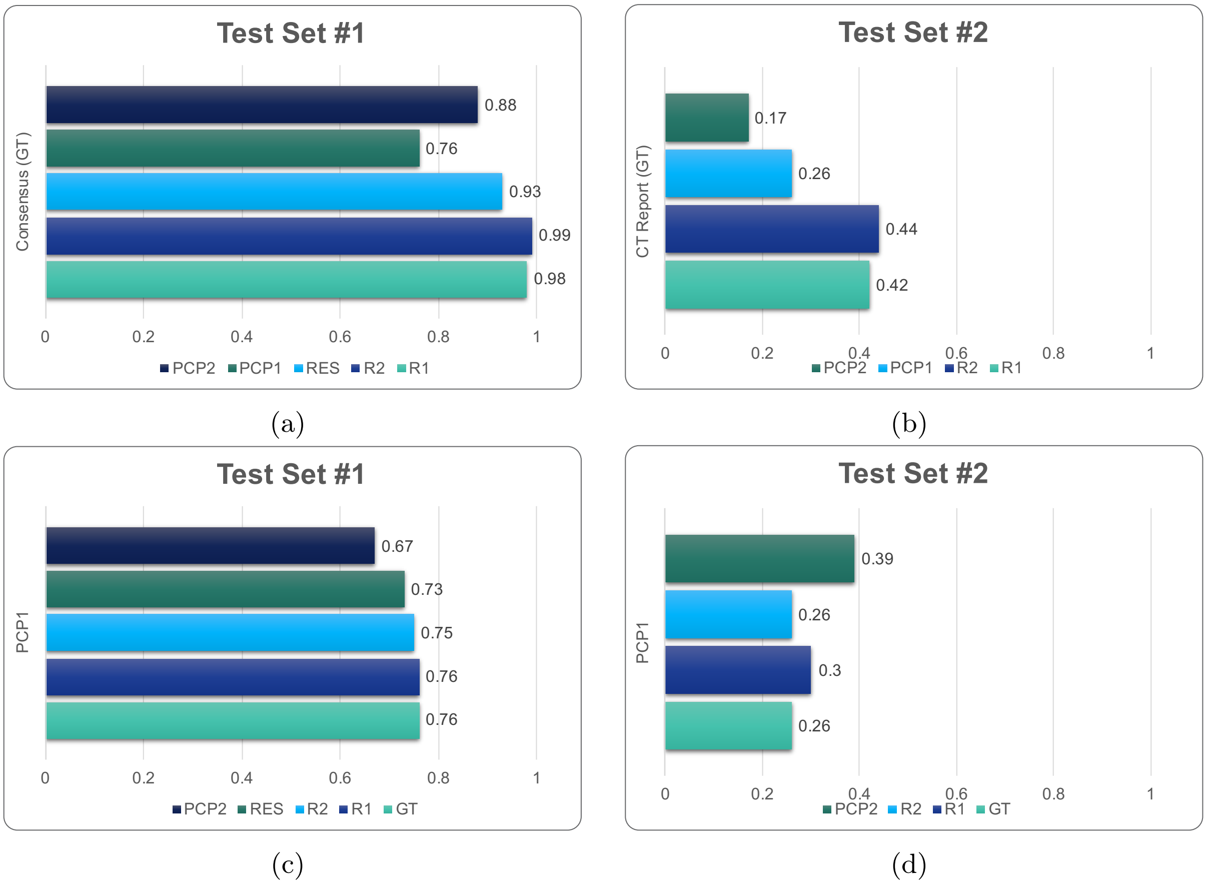

Figure S3. Inter-rater analysis using Cohen's Quadratic Kappa. (a) Agreement of Radiologist 1 (R1), Radiologist 2 (R2), Radiology Resident (RES), Primary Care Physician 1 (PCP1) and Primary Care Physician 2 (PCP2) with respect to the Ground Truth (GT) derived from consensus of two radiologists for the Test Set #1 (b) Agreement of R1, R2, PCP1 and PCP2 with respect to GT derived from CT report for Test Set #2. (c) Agreement of R1, R2, RES, PCP2 and GT with respect to PCP1 for Test Set #1. (d) Agreement of R1, R2, PCP2 and GT with respect to PCP1 for Test Set #2.

Model	LR	Momentum	Weight Decay	Nesterov
SeresNet50	$1e-1$, $1e-2$, $1e-3$,	0.0, 0.5, 0.9	0.0, $1e-3$, $1e-4$, $3e-4$,	Yes, No
Hourglass Net	$1e-1$, $1e-2$, $1e-3$,	0.0, 0.5, 0.9	0.0, $1e-4$,	Yes, No

Table S3. Hyperparameters search space. We kept the optimizer fixed to SGD. Batch size was 32 for SeresNet50 and 24 for the hourglass model (KNEEL[3])

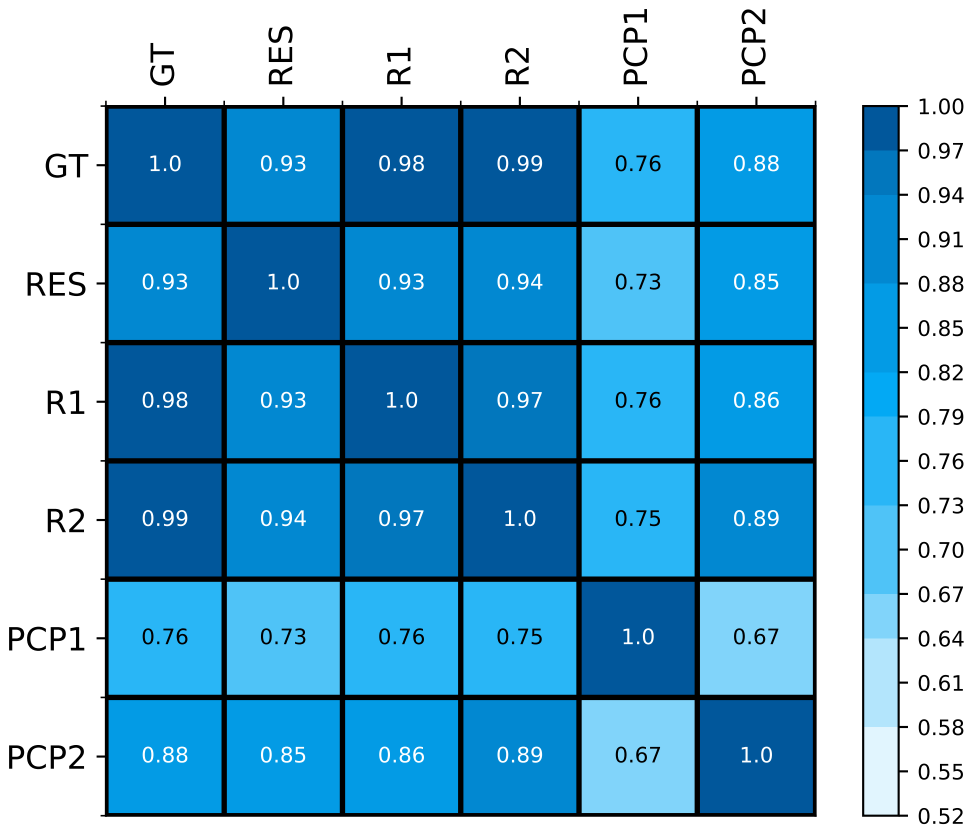

Figure S4. Cohen's Quadratic Kappa: all against all raters for Test Set #1

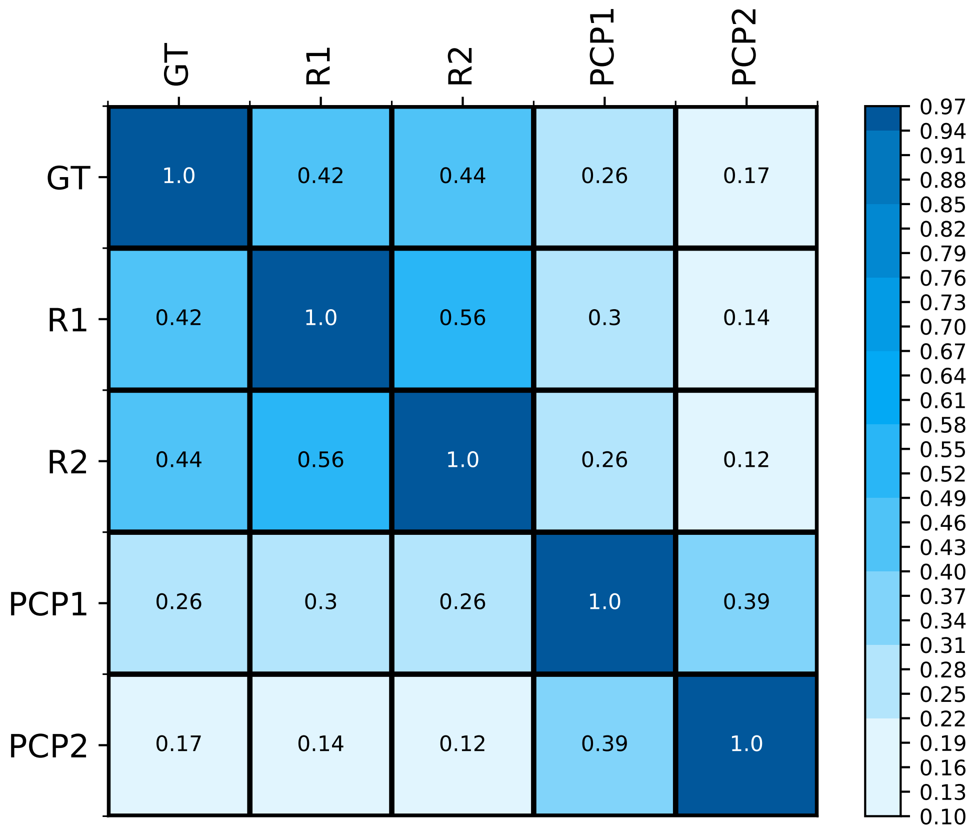

Figure S5. Cohen's Quadratic Kappa: all against all raters for Test Set #2

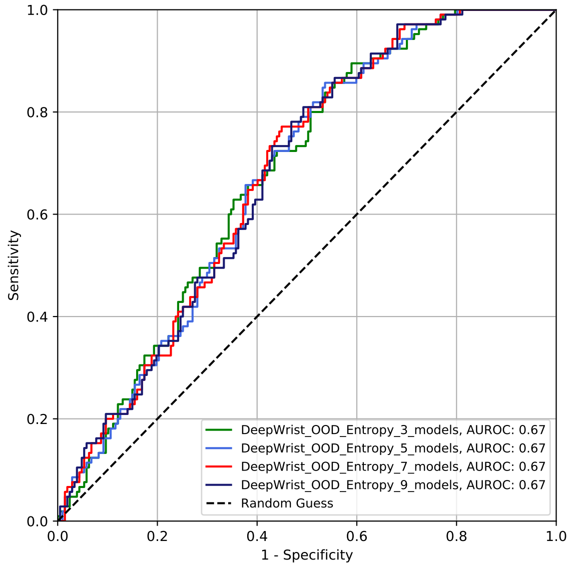

(a) AUROC

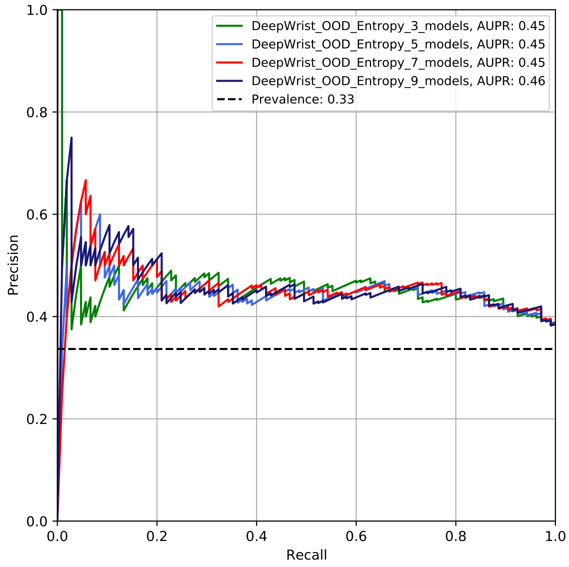

(b) AUPR

Figure S6. a) AUROC performance of OOD detection (by Entropy as uncertainty) using Deep Ensemble of 3,5,7 and 9 models respectively. b) AUPR performance of OOD detection for the same Deep Ensemble.

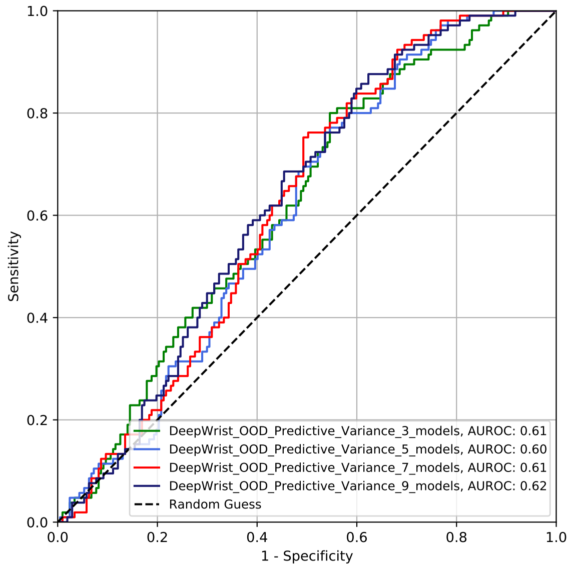

(a) AUROC

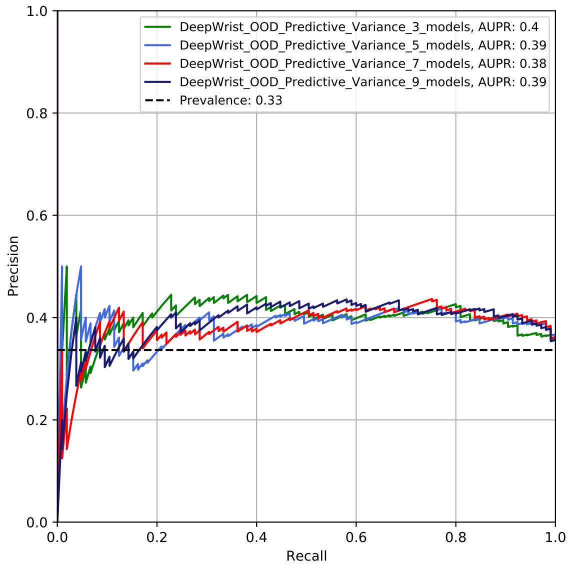

(b) AUPR

Figure S7. a) AUROC performance of OOD detection (by Predictive Variance as uncertainty) using Deep Ensemble of 3,5,7 and 9 models respectively. b) AUPR performance of OOD detection for the same Deep Ensemble.

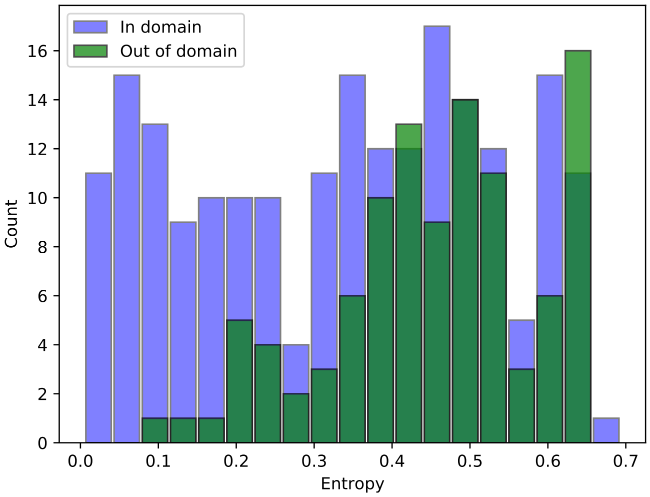

Figure S8. Entropy distribution of In-domain (general population cases) and Out-of-domain (challenging cases) data

References

1. Lakshminarayanan, B., Pritzel, A. & Blundell, C. Simple and scalable predictive uncertainty estimation using deep ensembles. In *Advances in neural information processing systems*, 6402–6413 (2017).

2. Guo, C., Pleiss, G., Sun, Y. & Weinberger, K. Q. On calibration of modern neural networks. *arXiv preprint arXiv:1706.04599* (2017).

3. Tiulpin, A., Melekhov, I. & Saarakkala, S. Kneel: Knee anatomical landmark localization using hourglass networks. In *Proceedings of the IEEE International Conference on Computer Vision Workshops*, 0–0 (2019).
